# Comparative adult preference–larval performance relationship between a specialist and a generalist tephritid: Implication for predicting field host‐range

**DOI:** 10.1002/ece3.70170

**Published:** 2024-08-13

**Authors:** Noémie Lauciello, Christian Gilbert Mille, Abir Hafsi, Vincent Jacob, Pierre‐François Duyck

**Affiliations:** ^1^ IAC, Equipe ARBOREAL La Foa New Caledonia; ^2^ CIRAD, UMR PVBMT Saint‐Pierre France; ^3^ CIRAD, UMR PVBMT Noumea New Caledonia

**Keywords:** *Artocarpus*, *Bactrocera tryoni*, *Bactrocera umbrosa*, bioecology, ecological niche, host‐fruits, invasion, New Caledonia, oligophagous insect, performance–preference, Tephritidae

## Abstract

Phytophagous insects differ in their degree of specialization to their host plants. It ranges from monophagous or oligophagous species that can only develop on a single host plant, or family of host plants, to extremely polyphagous species that can develop on plants from many distinct botanical families. The aim of this study was to compare the larval performance and adult preference of a highly generalist species, the Queensland fruit fly (*Bactrocera tryoni*) and a highly specialist species, the breadfruit fruit fly (*B. umbrosa*) among several fruits covering both species' host range. (i) larval performance was tested on 16 fruit species, and (ii) a female preference was tested on a subset of five fruit species. In addition, (iii) a field survey was carried out on 11 fruit species. *B. umbrosa* infested only *Artocarpus* fruits in the field. Accordingly, *B. umbrosa* larvae survived and developed only on fruits belonging to the *Artocarpu*s genus. Female *B. umbrosa* did not lay their eggs on non‐*Artocarpus* fruits, except *Terminalia catappa*. Female *B. tryoni*, on the other hand, made little selection between the fruits tested, and its larvae developed on 13 of the 16 fruit species tested. The larval performance of both species, adjusted when tested by female preference, predicted in large part the fruit infestation in the field. These data are essential to better estimate invasion risk where the species are not established.

## Graphical abstract

The aim of our study was to compare the larval performance and adult preference of a highly generalist species, *Bactrocera tryoni*, and a highly specialist species, *B. umbrosa*. Our data comfort the hypothesis that the preference–performance relationship is tighter in specialists than in generalist species. The larval performance of both species, adjusted when tested by female preference, predicted in large part the fruit infestation in the field.

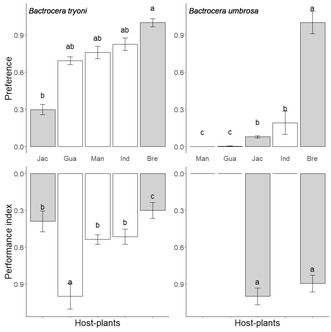

## 1 INTRODUCTION

Depending on the breadth of their host range, phytophagous insects can be placed on a continuum ranging from polyphagous species, known as “generalists,” capable of exploiting numerous plants belonging to a large number of botanical families to strictly monophagous species, known as “specialists,” capable of exploiting a single plant species (Jermy, 1984). At an intermediate level, “stenophagous” species can exploit few plant species only and “oligophagous” species might exploit several plants within a single botanical family. This continuum is called the degree of specialization. Defining the host range of a species might be somewhat tricky, since a plant can appear as a host in a given environment but not in another (Gomulkiewicz et al., 2000). The realized niche of a species in an environment is defined by such observations, as opposed to the more abstract concept of fundamental niche of a species which includes virtually all possible hosts in any environment (Hutchinson, 1957). The realized niche of a species in an environment from which it is absent may be predicted by the species' fundamental niche studied in controlled conditions (Vazquez, 2006). This is particularly useful in the context of biological invasion, where exotic species move to a different environment, the realized niche in the native environment may be restricted by abiotic factors such as interspecific competition (David et al., 2017).

In most insects, adults are more mobile than larvae, and larvae develop on a plant chosen by the adult. Specialization can therefore be divided into two categories (Ferry‐Graham, 2002; Irschick et al., 2005): physiological specialization, which results from physiological and morphological constraints that affect the insect's adaptation to the plant, that is performance (Futuyma & Moreno, 1988), and behavioral specialization, which results from behavioral constraints on host selection influencing the adult's choice of host plant, in other words, preference (Ferry‐Graham, 2002; Forister et al., 2012).

The breadth of an insect's host range depends on interactions with plants in the environment (which are modulated by phylogeny and host availability) as well as interspecific interactions with other insect species in the environment (Singer, 2008). These various factors contribute to narrowing the realized niche in an environment compared with the species' fundamental niche and might differ between geographically distinct areas (Gomulkiewicz et al., 2000). Thus, risk assessment of insect invasions needs a good understanding of fundamental niche and interspecific competition interactions (Clarke & Measham, 2022).

Despite numerous invasions of true fruit flies (Diptera: Tephritidae)that have already occurred and the determination to implement effective biosecurity measures, tephritids invasions are unlikely to stop, especially given the context of global change (Duyck et al., 2022; Papadopoulos et al., 2024; Trombik et al., 2023). In this family, species diet ranges from strict monophagy to high levels of polyphagy (Clarke, 2017). Immature tephritid larvae lack mobility outside the host fruit chosen by the female for oviposition, and therefore complete its development inside this fruit. A clear positive relationship between adult preference and larval performance in tephritids has been demonstrated for some species (Charlery de la Masselière, Ravigné, et al., 2017; Joachim‐Bravo et al., 2001; Joy Burrack & Zalom, 2008) but not for others (Balagawi et al., 2013; Birk & Aluja, 2018). The nutrient composition of host fruits is partly responsible for the adaptation of tephritid larvae to fruits (Hafsi et al., 2016; Raga et al., 2020); and increased specialization of generalist phytophagous tephritids when new invasive species arrive in their environment (Charlery de la Masselière, Facon, et al., 2017; Moquet et al., 2021). In addition, focusing on these preference–performance relationships provides good predictions of host plant use by tephritids in the field (Facon et al., 2021). However, the preference/performance relationship seems to be looser for those species with a wider host range. In particular, the following hypothesis has been suggested:

- Larval performance is a determining factor restricting the host range of specialist but not generalist species. Thus, larvae of generalist species should develop on a wide range of plants in laboratory experiments, whereas larvae of specialist species should be able to develop only on its host plants.
- The preference–performance relationship is tighter in specialist than generalist species. Indeed, females supposedly evolve to lay eggs on host plants which maximize the fitness of their offspring, leading to an optimal foraging strategy (Jaenike, 1978) also called *Mother know best hypothesis* (Gripenberg et al., 2010; Valladares & Lawton, 1991). A narrower range of plants suitable for larval development should induce stronger selection on females' host choice behavior.
- Studying larval development and female preference under controlled conditions enables predicting which fruits would be infested in the field. Generally speaking, insect species display a higher degree of specialization in the field than in laboratory conditions, and this may be due to other factors such as abiotic (climatic conditions) and/or biotic (interspecific competition, fly population size, adult mobility) (Facon et al., 2021). For introduced species that are experiencing a lesser effect from abiotic factors, that is, less or no interspecific competition in their novel environment than their native environment, studies under controlled conditions would be a more accurate depiction of how they will respond to a novel environment.

The aim of this study was to challenge these hypotheses by studying two species from the same genus and with more contrasting host range, specialist species of previous investigations developing on many species of the Cucurbitaceae family (Charlery de la Masselière, Facon, et al., 2017). The present study compares preference–performance relationships between a generalist species, the Queensland fruit fly, *Bactrocera tryoni* (Froggatt), and a specialist species, the Breadfruit fruit fly, *B. umbrosa* (Fabricius). *Bactrocera tryoni* has been considered the most abundant and problematic tephritid species in terms of damage in New Caledonia since its introduction in the late 1960s (Cochereau, 1970). Indeed, it is listed as a category A polyphagous fruit pest (Vargas et al., 2015) using 232 host species belonging to 49 different families (Hancock et al., 2000). *Bactrocera umbrosa*, native to East Asia and established in the Pacific including Papua New Guinea, the Solomon Islands, and Vanuatu (Krosch et al., 2019), is known to breed only on two fruit species belonging to the *Artocarpus* genus: breadfruit and jackfruit (Leblanc et al., 2013).

## 2 MATERIALS AND METHODS

### 2.1 Rearing of tephritids

This study of tephritids in New Caledonia included one polyphagous species (*B. tryoni*) and one oligophagous species (*B. umbrosa*). Laboratory colonies of *B. umbrosa* and *B. tryoni* were maintained from wild flies that emerged from jackfruit (*Artocarpus heterophyllus*) guava fruits (*Psidium guajava*), respectively, collected in South of New Caledonia in the La Foa area. The rearing room was fixed at 25 ± 1°C; 70 ± 15% relative humidity; L:D 12:12 photoperiod conditions, which allows the development of all studied species. Fruits were placed in boxes waiting for pupation and emergence of adults. The bottom of each box was covered with a layer of humidified Pinus sawdust to allow pupation of mature larvae. After emergence adults of each species were placed in rearing cages containing sugar, protein hydrolysate, and water. Eggs were collected from the adults' rearing cages using perforated plastic cups swabbed with the flesh of host fruits or artificial diets: breadfruit (*A. altilis*) for *B. umbrosa* and an artificial diet containing ripe banana puree, Torula yeasts (*Candida utilis*, H.J. Langdon) and methyl 4‐hydroxybenzoate (Nipagin, VWR International, BDH Chemicals) for *B. tryoni*. Eggs were placed in a Petri dish containing a humidified blotting.

### 2.2 Larval performance

Larval performances of *B. tryoni* and *B. umbrosa* have been measured from 16 host‐fruit species from 11 families (see Table 1). A simplified diet developed by Hafsi et al., 2016 was used. That diet contained 250 g of ripe fruit pulp without peel or seeds, 4 g of agar–agar (to provide a suitable texture), and 10 mL of a 4% Nipagin/sodium benzoate solution (to prevent fungal and bacterial growth). While these diets differed from fresh fruits in terms of physical texture, they allowed measuring individual fitness traits, following a high number of homogenous replicates, and obtaining comparable measurements of larval performance. Diet ingredients were blended together and placed in individual 5 mL plastic cups, each containing 5 g of diet mixture. Each combination of tephritid and fruit species was represented by 30 replicate cups, giving a total of 960 cups for the 16 fruits tested. One young larva (<2 h old after hatching) was placed carefully with a fine brush in each cup. No mortality due to physical handling was observed, as 100% survival was observed or the best host fruits. Each cup was then placed in the center of a larger container containing a thin layer of Pinus sawdust, allowing the larvae to settle after jumping out of the fruit‐based medium. The boxes were then closed using gauze and elastic to allow the larvae to breathe properly and avoid excess humidity. The boxes were then stored in a climate chamber (Memmert, HPP410ECO) with constant conditions (25 ± 1°C; 80 ± 1% relative humidity; L:D 12:12 photoperiod). Several indicators of larval performance were assessed in this study: Survival rate (mean number of larvae divided by the number of inoculated larvae across the 30 containers), developmental time, and pupal weight (Hafsi et al., 2016). Every 24 h until pupation, all cups were examined and pupae were collected. Larval survival was recorded as the number of pupae recovered from each host. Developmental duration was recorded as the time from placement in the cup to pupation. Each pupa was weighed with a precision scale (Kern EW220‐3NM, Kern & Sohn).

**TABLE 1:** Fruit species tested to study larval performance and field infestations of *Bactrocera tryoni* and *Bactrocera umbrosa*.

Family	Scientific name	Common name	Code
Anacardiaceae	*Mangifera indica*	Mango	Man^a^
Annonaceae	*Annona cherimola x Annona squamosa*	Atemoya	Ate
	*Annona muricata*	Soursop	Sou
	*Annona squamosa*	Custard Apple	Cus
Caricaceae	*Carica papaya*	Pawpaw	Paw
Combretaceae	*Terminalia catappa*	Indian almond	Ind^a^
Curcubitaceae	*Citrullus lanatus*	Watermelon	Wat
	*Cucumis melo*	Melon	Mel
	*Momordica charantia*	Bitter melon	Bit
Moreaceae	*Artocapus altilis*	Breadfruit	Bre^a^
	*Artocarpus heterophyllus*	Jackfruit	Jac^a^
Musaceae	*Musa spp*	Banana	Ban
Myrtaceae	*Psidium guajava*	Guava	Gua^a^
Oxalidaceae	*Averrhoa carambola*	Star fruit	Sta
Rubiaceae	*Coffea canephora*	Coffee bean	Cof
Rutaceae	*Citrus reticulata*	Mandarin orange	Ora

^a^ Indicates fruits used in the female choice experiments.

A larval performance index (survival rate x pupal weight/developmental duration) was then calculated in order to synthesize the three life‐history traits into a single datum, allowing easier comparisons with female preference and host specialization in the field.

### 2.3 Female preference

To assess the preference–performance relationship, we tested female preference of *B. umbrosa* and *B. tryoni* on five fruit species known to be good and widely distributed host plants of *B. tryoni*: two *Artocarpus* species, mango, Indian almond, and guava. Thirty naïve sexually mature (10–20 days old) females of each of the two species were placed in cages (dimensions 30 × 30 × 30 cm) containing proteins (Yeast hydrolysate enzymatic, MP Biomedicals), sugar, and water. Six egg‐laying devices consisting of a perforated (~84 holes) plastic cylinder with a lid (former photographic film boxes, dimensions 32 mm diameter, 52 mm high) filled with a piece of pulp of one of the five ripe fruit' species, or with a piece of humidified sponge (control) were randomly placed in each cage. A total of six replicates, each with 30 flies, was set up for each tephritid species. Eggs in each egg‐laying device were collected and counted after 24 h.

### 2.4 Host specialization in the field

Cultivated and wild fruits of the different studied species (Table 1) were randomly collected in cultivated fields, backyard gardens, and roadsides (fruits were collected from 02/20/2023 to 05/29/23). Number of fruits per sample varied with the fruit size and availability. Fruit samples were transported to the laboratory. The rearing room where the fruits were placed was at 25 ± 1°C; 70 ± 15% relative humidity; L:D 12:12 photoperiod conditions, which allows the development of all studied species. Fruit samples were weighed and placed individually in boxes for pupation. The bottom of each box was covered with a layer of humidified *Pinus* sawdust to allow pupation of mature larvae. Pupae were collected by sieving the sawdust once a week since fruit incubation. The pupae were weighed using a precision scale (Kern EW220‐3NM, Kern & Sohn), counted, and placed in small cages for emergence and identification.

### 2.5 Statistical analysis

Statistical analyses were performed using R‐4.1.0 software (R Development Core Team 2021). Larval survival was analyzed using a GLM (General Linear Model) with a binomial distribution as a function of host‐fruit species, fly species, and the interaction between these two factors. Larval development time, pupal weight, and larval performance index were treated by analysis of variance as a function of fruit‐host species, fly species, and the interaction between these factors.

The preference of females in the laboratory (expressed by the number of eggs laid in each fruit) was analyzed by a GLM with a Poisson distribution (deviance analysis with a quasi‐Poisson structure to account for over‐dispersion) as a function of species, host plant, and the interaction between these two variables.

Infestations in the field (expressed by the number of larvae per kg) were analyzed by a GLM with a Poisson distribution (deviance analysis with a quasi‐Poisson structure to account for over‐dispersion) as a function of species, host plant, and the interaction between these two variables.

Performance–preference relationships were analyzed using a linear model between larval performance indices and female choice rate on the same fruits, for each tephritid species.

## 3 RESULTS

### 3.1 Larval performance on different hosts

**FIGURE 1:**
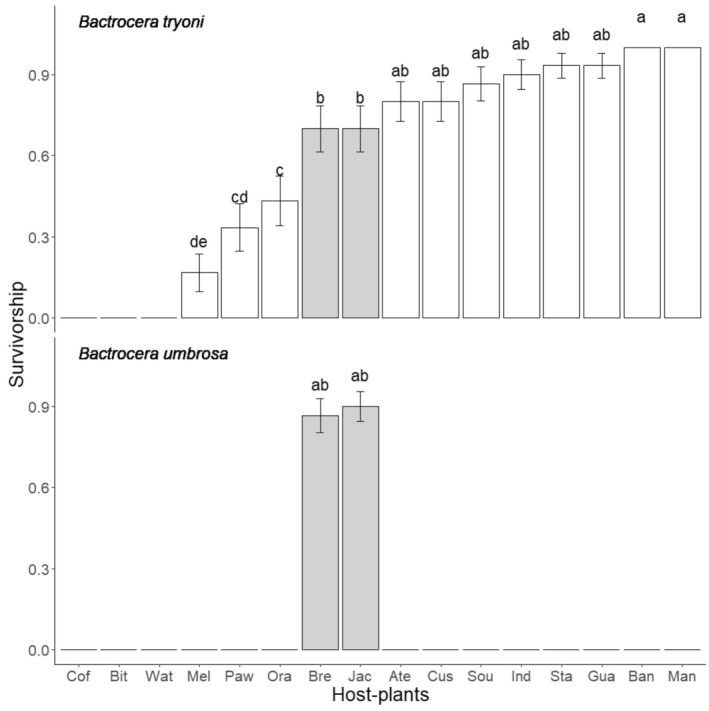
Larval survival rate (mean ± SE) for *Bactrocera tryoni* and *Bactrocera umbrosa* reared on 16 host plant species occurring in New Caledonia. Correspondence between the host plant species names and the three letter code can be found in Table 1. Gray bars correspond to fruits belonging to the *Artocarpus* genus. Means followed by different letters are significantly different. Kruskal–Wallis test with Bonferroni‐corrected pairwise comparisons (*p* < .05).

Larval survival rates were significantly different among fruit host species (ΔDev_15,927_ = 314, *p* < .001), *Bactrocera* species (ΔDev_15,927_ = 401, *p* < .001), and the interaction between these two factors (ΔDev_15,927_ = 176, *p* < .001). *Bactrocera tryoni* larvae were able to survive on a wide range of host fruits, surviving on 13 of 16 fruit species. The survival rate of *B. tryoni* was 100% for the banana and mango and was over 75% for six other fruit species (Figure 1). However, the observed survival rate was lower (around 70%) on the two *Artocarpus* fruit species, and even lower (less than 50%) on mandarin and papaya. Survival rate was low (<20% for melon) or null on the three Cucurbitaceae species tested, and no survival was also observed on coffee. *Bactrocera umbrosa* larvae survived only on the *Artocarpus* fruits studied (jackfruit and breadfruit) with a survival around 80%. No survival was observed on the remaining 14 fruit species (Figure 1).

**FIGURE 2:**
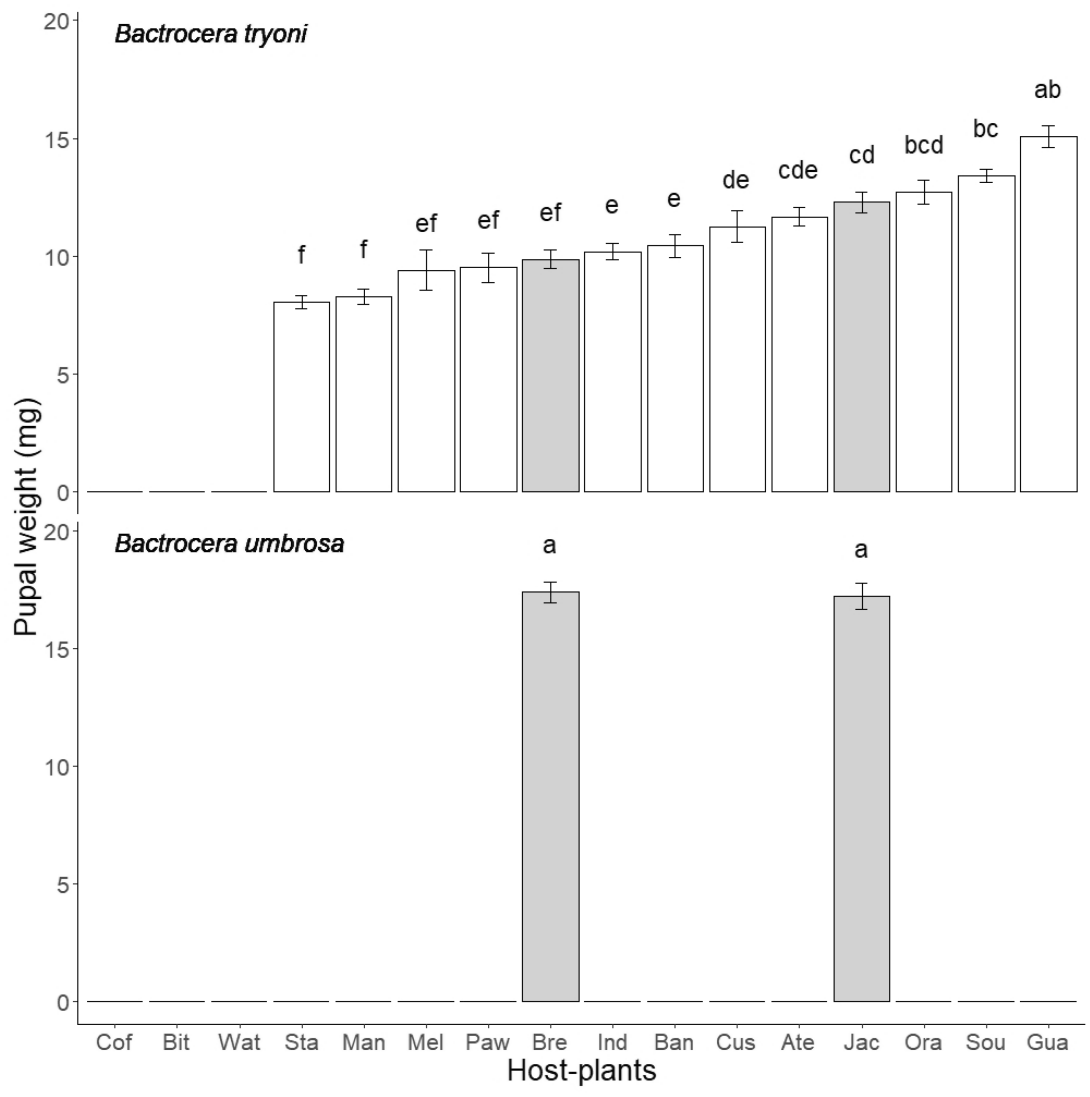
Pupal weight (mean ± SE) for *Bactrocera tryoni* and *Bactrocera umbrosa* reared on 16 host plant species occurring in New Caledonia. Correspondence between the host plant species names and the three letter code can be found in Table 1. Gray bars correspond to fruits belonging to the *Artocarpus* genus. Means followed by different letters are significantly different. Kruskal–Wallis test with Bonferroni‐corrected pairwise comparisons (*p* < .05).

Pupal weights differed significantly between fruit‐host species (F_12,329_ = 35, *p* < .001), *Bactrocera* species (F_1,329_ = 192, *p* < .001), and the interaction between these two factors (F_1,329_ = 8, *p* = .004) (Figure 2). *Bactrocera tryoni* is heavier on guava (15 mg), followed by soursop (13 mg), jackfruit (12 mg), and mandarin (12 mg). *Bactrocera tryoni* were lighter on carambola and mango (8 mg) with no significant difference between the two (Kruskal–Wallis test with Bonferroni‐corrected pairwise comparisons *p* > .05). *Bactrocera umbrosa* pupal weights were equivalent on the two *Artocarpus* species it survived in (superior than 15 mg), while this parameter could not be measured in the other fruit species.

**FIGURE 3:**
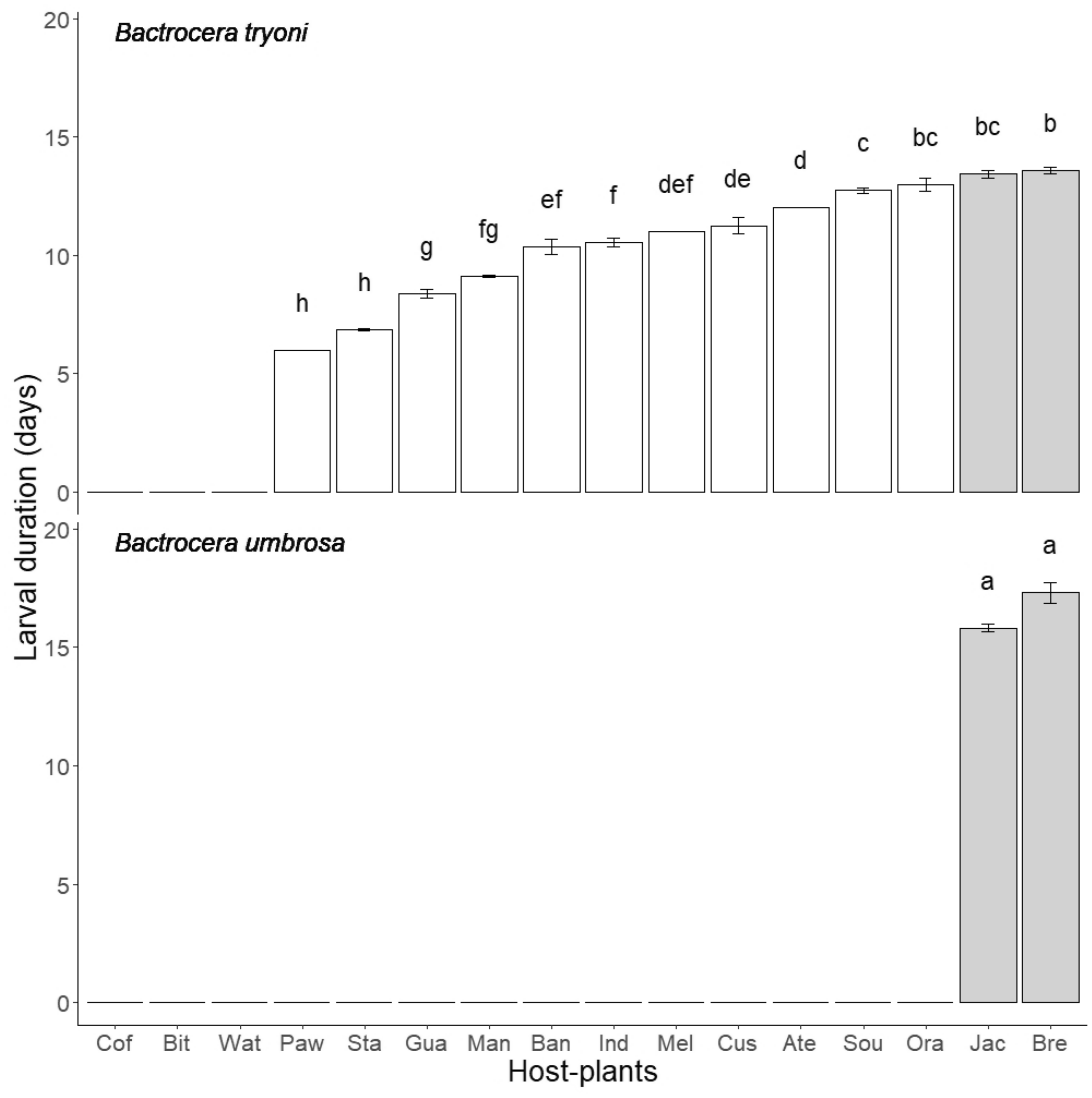
Development duration (mean number of days between hatching and pupation± SE) for *Bactrocera tryoni* and *Bactrocera umbrosa* reared on 16 host fruit species occurring in New Caledonia. Correspondence between the host‐plant species names and the three letter code can be found in Table 1. Gray bars correspond to fruits belonging to the *Artocarpus* genus. Means followed by different letters are significantly different. Kruskal–Wallis test with Bonferroni‐corrected pairwise comparisons (*p* < .05).

Larval development durations were significantly different between host‐fruit species (F_12,325_ = 188, *p* < .001), *Bactrocera* species (F_1,325_ = 176, *p* < .001), and the interaction between these two factors (F_1,325_ = 9, *p* = .004) (Figure 3). *Bactrocera tryoni* developed the quickest on papaya and carambola (less than 7 days for both fruits) and the longest in the two *Artocarpus* fruits (more than 15 days for both fruits). (*Bactrocera umbrosa* showed no significant difference between the two *Artocarpus* fruit species more than 15 days for both).

### 3.2 Female preference and preference–performance relationship

**FIGURE 4:**
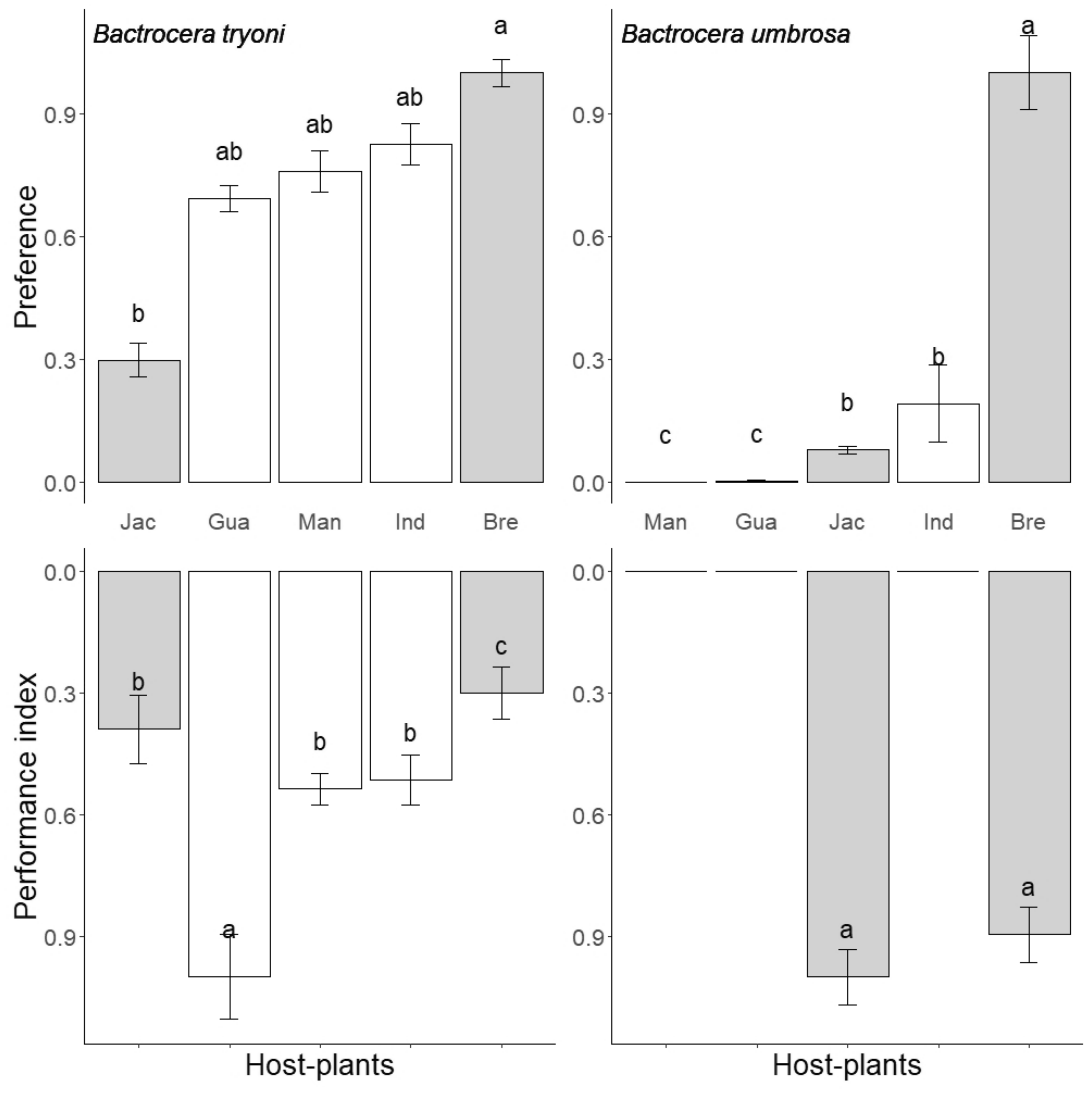
Female (mean number of eggs laid ± SE) and larval performance (mean for *Bactrocera tryoni* and *Bactrocera umbrosa* species on five host plant species occurring in New Caledonia). For better visualization values are expressed relative to the maximum value for each graph. Correspondence between the host‐plant species names and the three letter code can be found in Table 1. Gray bars correspond to fruits belonging to the *Artocarpus* genus. Means followed by different letters in the same panel are significantly different. Kruskal–Wallis test with Bonferroni‐corrected pairwise comparisons (*p* < .05).

For all experiments on female preference, the number of eggs laid was significantly different between tephritid species (ΔDev_1,50_ = 5597, *p* < .001), host species (ΔDev_4,50_ = 1279, *p* < .001), and the interaction between these two variables (ΔDev_4,50_ = 918, *p* = .026) (Figure 4). Analyzing the two *Tephritidae* species separately, we note that the host plant species had a marked effect on the choice of *B. umbrosa* females (ΔDev_5,25_ = 1372, *p* < .001), while this variable had overall less effect on the choice of *B. tryoni* females (ΔDev_4,25_ = 826, *p* = .242). *Bactrocera tryoni* females preferred laying eggs on breadfruit, followed by Indian almond, almond mango, and guava, with no significant difference among these four fruits (Wallis test with Bonferroni‐corrected pairwise comparisons, *p* = .12). Bactrocera tryoni oviposited in all host‐fruit species in this experiment (Figure 4).

For *B. umbrosa* there was a significant linear correlation between female preference and larval performance (*y* = 0.680*x* + 0.23, *R* ^*2*^ = .260, *p* = .004), while the relationship was not significant for *B. tryoni* (*y* = 0.035 *x* + 0.56, *R* ^*2*^ = .001, *p* = .85).

### 3.3 Realized and fundamental niches comparison

**FIGURE 5:**
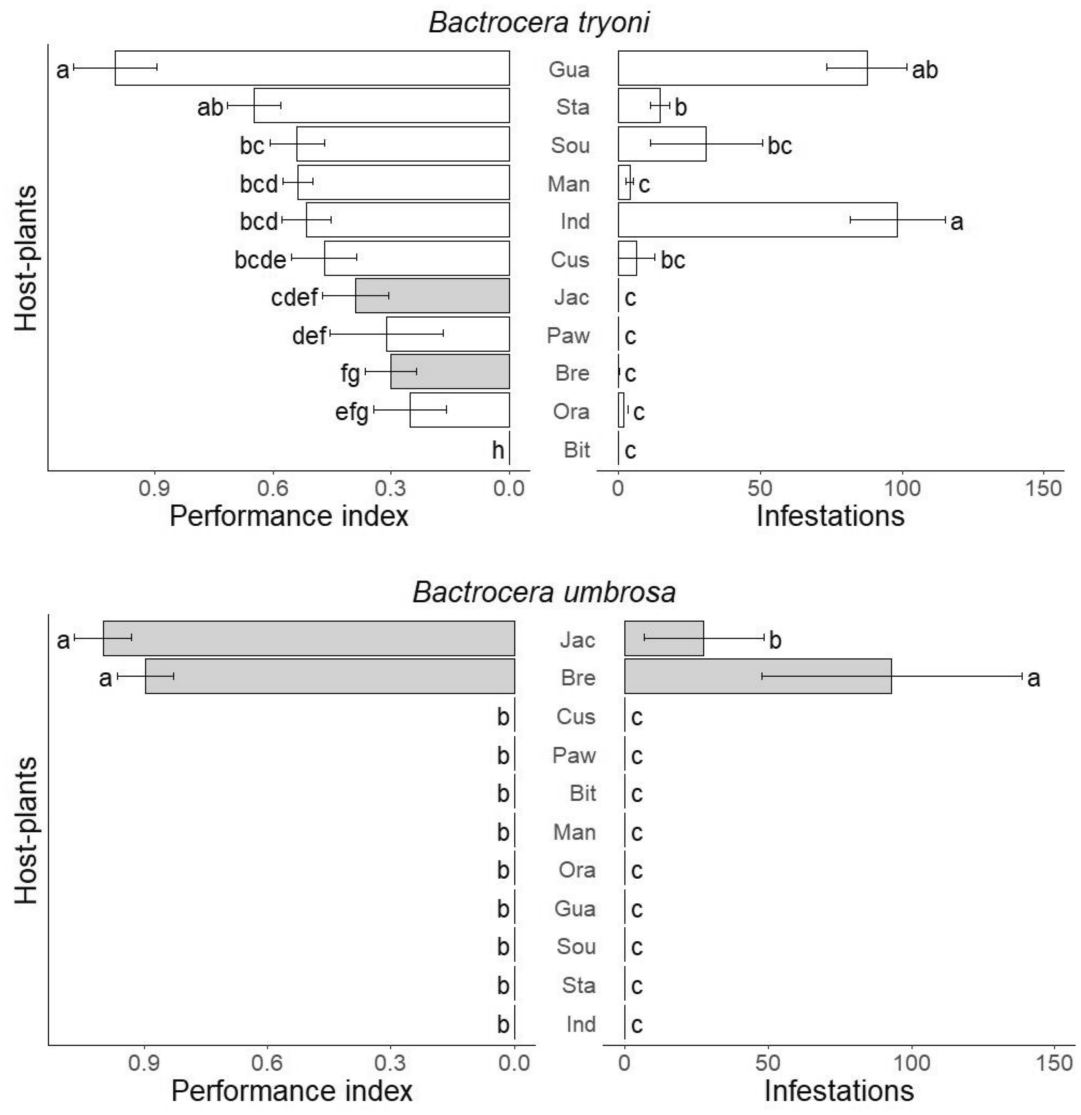
Performance index (mean) of *Bactrocera tryoni* and *Bactrocera umbrosa* larvae and field infestations (mean number of pupae per kg ± SE) on 11 host fruit species occurring in New Caledonia. For better visualization values of performance are expressed relative to the maximum value for each graph. Correspondence between the host‐plant species names and the three letter code can be found in Table 1. Gray bars correspond to fruits belonging to the *Artocarpus* genus. Means followed by different letters in the same panel are significantly different. Kruskal–Wallis test with Bonferroni‐corrected pairwise comparisons (*p* < .05).

The larval performance index was significantly different between host fruit species (F_10,638_ = 39, *p* < .001), *Bactrocera* species (F_1,638_ = 58, *p* < .001), and the interaction between these two factors (F_10,638_ = 57, *p* < .001) (Figure 5). Guava showed the best larval performance for *B. tryoni* with star fruits followed by soursop, mango, Indian almond, and custard apple with equivalent performance (Figure 5). For *B. tryoni*, *Artocarpus* fruits provide moderate performance. The performance of *B. umbrosa* is slightly better on breadfruit than on jackfruit, while the difference was not significant (Figure 5).

Only jackfruit and breadfruit were infested by *B. umbrosa* in the field with an average of 27 and 93 pupae per kg of fruit for, respectively (Figure 5). *Bactrocera umbrosa* specialization in the field is consistent with performance, with no significant difference between jackfruit and breadfruit. For *B. tryoni*, the most infested fruits were guava and Indian almond with an average of 88 and 98 pupae per kg of fruit, respectively. However, there is a difference between these two fruits in terms of performance for the larvae. Guava is the fruit with the best performance for *B. tryoni* larvae, while Indian almond fruit does not stand out from the other fruits tested (Figure 5). Conversely, mandarin orange showed a lower infestation, with around 2 pupae per kg of fruit, despite a high performance in terms of pupal weight (cf. Figure 2).

## 4 DISCUSSION

### 4.1 Larval development under controlled conditions enables predicting fruit infestations in the field

The obtained results provide a clear picture of performance and preference of a generalist and a specialist *Tephritidae* via infestation analysis. Indeed, we found that 1 kg of jackfruit and breadfruit contains an average of 27 and 93 pupae of *B. umbrosa*, respectively. While *B. umbrosa* shows high larval performance for jackfruit and breadfruit in the laboratory, breadfruit seems to be the most favored resource for the females to lay their eggs. Thus, fruit infestations in the field appear to be predicted by both larval performance and adult preference.

Regarding *B. tryoni*, the most infested fruits in the field were guava (*Psidium guajava*) and Indian almond (*Terminalia catappa*), with an average of 88 and 98 pupae per kg of fruit, followed by soursop (*Annona muricata*) and with 31 larvae per kg of fruit. Guava is the fruit showing both the best larval development in the laboratory and the highest infestations in the field.

Both *B. tryoni* and *B. umbrosa* laid more eggs on Indian almonds than on other fruits. However, no larval development of *B. umbrosa* was observed in this fruit, neither in the laboratory nor in the field. Indian almond is known to be a host fruit of numerous polyphagous *Bactrocera* species (Charlery de la Masselière, Ravigné, et al., 2017; Moquet et al., 2021). One of the compounds known from Indian almonds is methyl eugenol (Siderhurst & Jang, 2006), which plays a role in sexual communication in several *Bactrocera* species (Starkie et al., 2022). *Bactrocera umbrosa* males are attracted to methyl eugenol and consume it, which in turn improves their propensity to attract females and their mating success (Wee et al., 2018). While essentially a male attractant, methyl eugenol is regularly thought to have a behavioral role for females (Raghu, 2004), which might explain why *B. umbrosa* lays preferentially its eggs in this fruit.

Star fruit (*Averrhoa carambola*) is, for its part, subject to lower infestations, with 14 larvae per kg of fruit, but it is the fruit that provides the best resources for larvae after guava in the present study. In general, specialization is more obvious in the field, which may be due to other factors such as abiotic (climatic conditions) and/or biotic factors (interspecific competition, fly population size, adult mobility) (Facon et al., 2021).

### 4.2 Interspecific competition in the field

The results suggest that *Artocarpus* are potential host for these two *Tephritidae* species. In fact, both species survived on the two fruits, jackfruit was one of the host fruits with the highest pupal weight for *B. tryoni*, and breadfruit was preferred by adult females of the two species over guava, mango, and Indian almond in our bioassay. However, analysis of infestations in the field did not reveal the presence of *B. tryoni* in *Artocarpus* fruits. This phenomenon could be linked to a better performance of *B. umbrosa* in these fruits, and therefore potentially a better ability to compete with other tephritid species within these fruits. In French Polynesia, where *B. umbrosa* is absent, *B. dorsalis* and *B. tryoni* mainly exploit breadfruit, and in Micronesia, *B. frauenfeldi* also uses this host fruit. However, in countries where *B. umbrosa* and *B. tryoni* are both established, such as New Caledonia, *B. umbrosa* is thought to out‐compete *B. tryoni* for *Artocarpus* resources (Leblanc et al., 2013). The quantity of alternative resources defining the degree of specialization may play a role in the insects' investment in competition. Indeed, specialists, which are better adapted to a restricted host range (in our case, *Artocarpus*), will have few or no plants on which to take refuge and will have a greater investment in competition to exploit this resource (Bili et al., 2016). Conversely, generalists are more effective at avoiding competition than specialists. In the presence of competitors, generalists will instead opt for flight behavior and change their resource‐use behavior, mainly by seeking alternative resources or increasing their host range (Denno et al., 1995). Anyway, we can conclude that regarding host fruits in New Caledonia, the realized niche of *B. umbrosa* is close to its fundamental niche, while the realized niche of *B. troni* is more restricted than its fundamental niche.

### 4.3 Larvae of generalist species show a high performance in a wide host‐plant range in laboratory experiments compared to the specialist species

The results showed that larvae from a generalist species, *B. tryoni*, were able to survive and grow on a wide host species range. This kind of strategy allows them to have a performance almost uniform on all resources but a fitness that is rarely optimal. *Bactrocera tryoni* is even able to develop on species that is not part of its host range such as jackfruit, with a higher pupal weight on this fruit than on some of its host fruits such as mango and starfruit. Jackfruit contains more carbohydrates compared to these two other species (USDA, National Nutrient Database for Standard Reference, and ANSES French Agency for Food, Environmental and Occupational Health Safety, databases). This reflects also strong phenotypic plasticity during larval development, facilitating adaptation to distinct host plants. For the specialist *B. umbrosa*, larvae developed and showed optimum performance on a limited part (only *Artocarpus* spp.) of the range of fruit tested. This suggests that the two tephritids species tested differ in terms of nutrient requirements. For example, *Artocarpus* fruits have the highest potassium contents of the tested fruits (490 and 448 mg/100 g of fruit for breadfruit and jackfruit, respectively), (USDA, National Nutrient Database for Standard Reference, and ANSES French Agency for Food, Environmental and Occupational Health Safety, databases) and breadfruit contains a large amount of starch (20 g/100 g of fruit) (Huang et al., 2000). It would be interesting to analyze in greater detail the correlation between larval development and the biochemical composition of the 16 fruits tested. In a study of the host plant range of fruit fly populations in La Réunion, the performance of polyphagous species was strongly associated with carbohydrate, lipid, and fiber contents and was not associated with protein content (Hafsi et al., 2016). It has been suggested by Behmer (2009) that some phytophagous individuals prefer diets rich in sugars, while other species show a preference for diets with high levels of protein. In addition to these nutrients, toxic elements, secondary metabolites as well as fruit characteristics like texture can exert an influence on larval performance (Bateman, 1972). While we studied performance on larval stages which are directly affected by fruit species and composition, other stages may be affected by the fruit species and may be important for overall species fitness. For example, *B. tryoni* is able to compensate fitness loss by high fecundity (Balagawi et al., 2023). Also, we tested larval development in fruits using diets with fruit pulp, but in fruits such as banana, watermelon, or melon, the thickness and hardness of the pericarp may create a mechanical barrier for female egg laying despite the fact that the flesh of these fruits is suitable for *B. tryoni* larval development.

### 4.4 Preference–performance relationship is stronger in specialists than generalist species

Charlery de la Masselière et al. (2017) showed that the preference–performance relationship is closer in cucurbit specialist tephritids than in generalist species. The data presented in our study allow us to generalize this observation to specialist tephritids because we demonstrate the same phenomenon with two species, differing in their degree of specialization but within the same genus *Bactrocera*, thus with a more recent evolutionary divergence than what was previously shown. According to the *Mother knows best* hypothesis, female phytophagous insects evolved to lay eggs on plants with optimal quality for the development of their offspring (García‐Robledo & Horvitz, 2012; Gripenberg et al., 2010). Given that plants possess uneven and variable nutritional qualities, females ovipositing on hosts offering the necessary resources to enable optimal larval development would maximize their selective value. This evolutionary mechanism should result in a positive relationship between preference and performance (Gripenberg et al., 2010; Jaenike, 1978). However, being selective is costly in terms of time and energy consumption due to locomotor activity (Janz, 2003), and also in terms of energy consumption due to the neuronal processing of sensory information (Niven & Laughlin, 2008). The cost of host selection on fitness is stronger for generalist species than for specialist species (Bernays, 2001; Cunningham, 2012), and could balance the benefits of a positive preference–performance relationship. The differential preference–performance relationship we observed between a specialist and a generalist species could result from such trade‐off.

Richards et al. (2006) described two opposite ecological strategies that could promote a species' adaptation to a new environment and thereby invasions. The *Master of some* strategy applies to species with a narrow ecological niche for which the species has a high degree of fitness, outperforming competitors. The *Jack of all trades—master of none* strategy consists of species with a plastic ecology, that is able to develop on a diversity of ecological conditions, for which it does not need an outstanding fitness. The invasive success of such species derives from a relatively constant selective value in the face of changing environments. Our data suggest that the performance/preference relationships of *B. umbrosa* and *B. tryoni* comply with the first and second strategies, respectively. In *B. umbrosa*, females oviposit essentially on the few fruits which promote good larval development, and this ensures a good fitness on a narrow niche. In *B. tryoni*, the fitness is not as high as *B. umbrosa* on each fruit considered individually, due to a looser preference/performance relationship, but the invasive success of the species is ensured by the diversity of fruits included within its wide host range.

### 4.5 Implications for biosecurity and pest risk analysis

Understanding the processes by which phytophagous insects interact with new host plants is particularly important for predicting and preventing invasions. All these results show the usefulness of studying larval development under controlled conditions for estimating the species' fundamental niche, in order to make predictions regarding the species' realized niche in the field. For estimating invasion risk in the Tephritidae family, data on associations with host plants are essential. Most of the time, field surveys are used to estimate the host status, however host status in the field is influenced by tephritid population levels, interspecific competition among tephritids and abiotic factors of the environment (Clarke & Measham, 2022; Duyck et al., 2006; Facon et al., 2021). The very close association shown in our study between *B. umbrosa* and *Artocarpus* species in both fundamental niche and realized niche suggests that if this species acquires a new host plant, it would most likely be closely related to the *Artocarpus* genus. Thus, surveying plants phylogenetically related to *Artocarpus* in new habitats and regions would help predict their susceptibility to invasion by *B. umbrosa* and allow an early detection of this pest. This can be directly applied for biosecurity in the East Pacific where *B. umbrosa* is not present, while breadfruit is very important for food security and culture conservation in the communities.

*Bactrocera tryoni is* an important invasive species that has already invaded New‐Caledonia and French Polynesia but might invade in the near future many territories in the Pacific where it is absent (Duyck et al., 2022), but also Europe where it is quarantine species. In the case of generalist species such as *B. tryoni*, biochemical composition such as sugar content is a better indicator (Hafsi et al., 2016) than host fruit phylogeny for predicting whether new fruits would be potential hosts. A better understanding of species fundamental host range for species at risk of introduction in New Caledonia such as *Bactrocera dorsalis* and *Zeugodacus curbubitae* (Duyck et al., 2022) would also contribute to improving Biosecurity procedures.

## AUTHOR CONTRIBUTIONS

**Noémie Lauciello:** Conceptualization (equal); data curation (lead); formal analysis (supporting); investigation (equal); methodology (lead); writing – original draft (equal); writing – review and editing (equal). **Christian Gilbert Mille:** Conceptualization (equal); funding acquisition (lead); methodology (equal); project administration (equal); supervision (lead); writing – original draft (equal); writing – review and editing (equal). **Abir Hafsi:** Methodology (equal); validation (equal); writing – review and editing (equal). **Vincent Jacob:** Validation (equal); writing – original draft (equal); writing – review and editing (lead). **Pierre‐François Duyck:** Conceptualization (lead); data curation (equal); formal analysis (lead); funding acquisition (equal); methodology (equal); supervision (lead); writing – original draft (lead); writing – review and editing (lead).

## CONFLICT OF INTEREST STATEMENT

The authors declare that there are no conflicts of interest.

## Data Availability

All relevant data are within the paper and its Supporting Information section.
